# A Novel Ultrasonic Welding‐Assisted Thermoplastic Root Canal Obturation Technique: An Ex Vivo Proof‐of‐Concept Study

**DOI:** 10.1002/cre2.70356

**Published:** 2026-04-17

**Authors:** Mohammed Alshehri, Chaniotis Antonis, Hadi Alamri, Ahmad AlQahtani, Mohammed Altamimi

**Affiliations:** ^1^ Center of Excellence in Biotechnology Research & King Saud University Medical City Riyadh Riyadh Region Saudi Arabia; ^2^ National and Kapodistrian University of Athens Athens Greece; ^3^ Department of Dentistry King Faisal Specialist Hospital and Research Centre Riyadh Riyadh Region Saudi Arabia; ^4^ Riyadh Specialized Dental Center Second Health Cluster, Ministry of Health Riyadh Riyadh Region Saudi Arabia; ^5^ Dental University Hospital King Saud University Medical City Riyadh Riyadh Region Saudi Arabia

**Keywords:** endodontics, gutta‐percha, root canal therapy, thermoplastic obturation, ultrasonic welding

## Abstract

**Objectives:**

To evaluate the effectiveness of an ultrasonic welding (USW)‐assisted thermoplastic obturation technique for improving root canal sealing, void reduction, and lateral canal penetration.

**Methods:**

A three‐phase experimental approach was employed: (1) Customized gutta‐percha points were developed, optimized to incorporate ultrasonic‐responsive thermoplastic obturation cores; (2) in vitro testing was conducted using micro‐computed tomography (CT) and infrared thermometry to assess lateral canal flow and thermal safety, respectively; and (3) an ultrasonic generator (28+ kHz), booster, and sonotrode were used to generate the heat required for obturation.

**Results:**

Compared with traditional methods, the USW technique demonstrated a mean void reduction of 62% (95% CI: 58%–66%, *p* < 0.001), significant lateral canal penetration, and maintained temperatures safely below 45°C. No microfractures were observed in the treated teeth.

**Conclusions:**

The USW‐assisted obturation technique facilitates and enhances root canal obturation, reduces voids, and maintains tooth structural integrity through precise energy application combined with improved flow mechanics.

## 1 Introduction

Endodontic obturation ideally aims to completely seal the root canal system in all dimensions, thereby preventing reinfection and ensuring long‐term success. Owing to the failures of conventional techniques in filling complex anatomies, i.e., lateral canals, isthmuses, and micro irregularities (Gasner and Brizuela 2025), existing techniques should be improved. Ultrasonic welding (USW), an otherwise industrial process, relies on the use of high‐frequency mechanical vibrations to soften and merge thermoplastic materials (Bhudolia et al. 2020). Applications in biomedical fields, such as orthopedic implants and cranial fixation, have demonstrated the precision, efficiency, and safety of this technique (Alkahtany et al. 2024; Pietrzak 2025; Sharki and Ali 2024). Despite advances in materials and techniques, incomplete obturation and the presence of voids remain common challenges, which can compromise long‐term treatment outcomes (Mehta et al. 2025).

Recently, ultrasonic energy has been explored in endodontics to enhance the flow and adaptation of obturation materials (Shin et al. 2025). Ultrasonic‐assisted methods can generate localized heat, improve the plasticity of thermoplastic materials, and promote deeper penetration into lateral canals without compromising tooth structure (Mozo et al. 2012). Conventional methods, such as warm vertical compaction and single‐cone techniques, often leave voids and fail to completely fill complex canal anatomies (Jaha 2024).

USW technology exhibits suitable dovetailing prospects in the field of endodontics to meet the challenge of sealing the root canal system. This preclinical study introduces the use of USW technology in endodontic obturation using bovine teeth as a translational model. In this novel obturation method, ultrasonic energy is used to selectively soften and adapt thermoplastic gutta‐percha points in situ. By modifying the viscosity of the material on demand, this approach aims to enhance flow into often problematic and challenging lateral extensions of the root canal system while avoiding excessive heat or apical extrusion. The objective of this study is to evaluate the effectiveness of the USW‐assisted obturation technique, providing a foundation for its potential application in clinical endodontics.

## 2 Methods

### 2.1 Study Design

This proof‐of‐concept ex vivo experimental evaluation study was conducted to assess the feasibility, safety, and working mechanism of a novel ultrasonic welding (USW)‐assisted thermoplastic obturation technique.

### 2.2 Ex Vivo Experimental Procedures

All procedures followed institutional guidelines for the handling of animal‐derived tissues. The study included three sequential phases. **Phase I** involved feasibility studies using flat models simulating lateral canals, in which gutta‐percha pins (types I and II) were ultrasonically activated for 2–8 s, and deformation and flow patterns were recorded using transparent obturator core models I and II. **Phase II** involved optimization of the ultrasonic system, including calibration of amplitude and power for safe intraoral use while achieving successful manipulation of gutta‐percha. **Phase III** involved ex vivo testing on bovine teeth, which were decoronated, instrumented, and obturated using ultrasonically activated gutta‐percha. Micro‐computed tomography (micro‐CT) scans were obtained before and after obturation to quantify filling effectiveness and sealing quality.

### 2.3 Sample Selection

A total of 30 extracted bovine teeth were used, divided into two sets of 15 teeth each. Teeth with cracks, resorption, prior endodontic treatment, or other structural defects were excluded. All specimens were stored in 0.1% thymol solution at 4°C until experimentation.

Bovine teeth were selected as experimental models due to their structural and compositional similarity to human dentin, particularly in terms of mineral content, tubule orientation, and mechanical properties. In addition, they provide larger root dimensions, allowing standardized preparation and obturation while minimizing variability across specimens. The use of bovine teeth instead of human teeth in research is also recommended in many studies. The use of bovine teeth instead of human teeth in research has also been recommended in many studies (Al Ankily et al. 2025; Franchini Pan Martinez et al. 2023).

To evaluate reproducibility, the experiment was performed in two independent rounds of testing. Each round included 15 bovine teeth prepared and obturated using the same ultrasonic welding‐assisted protocol. The second round of testing was conducted by different researchers under identical experimental conditions to assess operator‐independent reproducibility.

### 2.4 Root Canal Preparation

Teeth were decoronated to a standardized length, and working length was established 1 mm short of the apex using a size #10 K‐file. Canals were prepared using HyFlex CM and EDM rotary files. Irrigation was performed using 2.5% sodium hypochlorite, followed by a final rinse with 17% EDTA and distilled water.

### 2.5 Root Canal Material Removal

To simulate retreatment and ensure standardized starting conditions, all canals were initially obturated with conventional gutta‐percha and sealer using lateral condensation. Existing obturation material was removed using rotary retreatment files and ultrasonic tips. Complete removal was confirmed visually under a dental operating microscope and with micro‐CT imaging.

### 2.6 USW‐Assisted Thermoplastic Obturation

**Figure 1:**
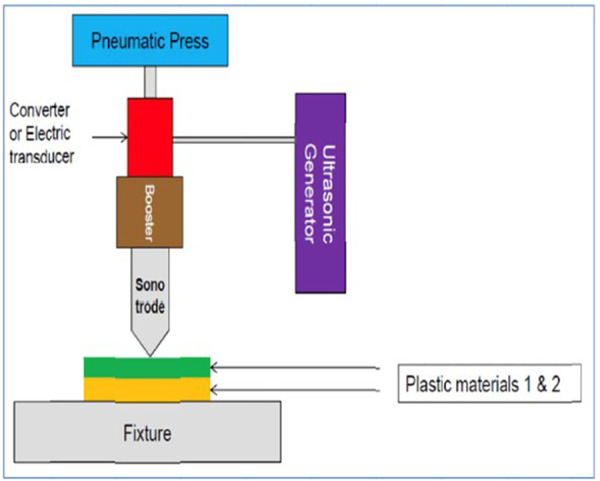
Setup of the ultrasonic welding‐assisted obturation system, including the generator (electrical signal source), booster (amplitude modifier), and sonotrode (high‐frequency vibration transmitter).

**Figure 2:**
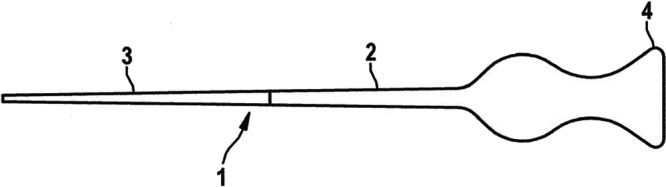
Side view of the obturation point showing: (1) endodontic obturation point, (2) core material, (3) outer material, and (4) coupling element (handle).

Ultrasonic generator (Sonics & Materials Vibra‐Cell VCX 500, USA; ≥ 28 kHz) was equipped with a booster and titanium sonotrode (tip diameter 2 mm) (Figure 1). Customized gutta‐percha cones with deformable thermoplastic cores were developed (Figure 2). The core is a polymer composition based on gutta‐percha, formulated with specific additives to optimize its acoustic impedance and viscoelastic response for ultrasonic activation. Setup of the ultrasonic welding‐assisted obturation system, including the generator (electrical signal source), booster (amplitude modifier), and sonotrode (high‐frequency vibration transmitter). Based on Phase II optimization, generator power was set at 50% (50 W), with continuous activation for 4–6 s per tooth. The sonotrode was applied directly to the gutta‐percha within the canal using light axial pressure (~10 N). Infrared thermometers monitored temperature in real time, maintaining safety below 45°C.

**Figure 3:**
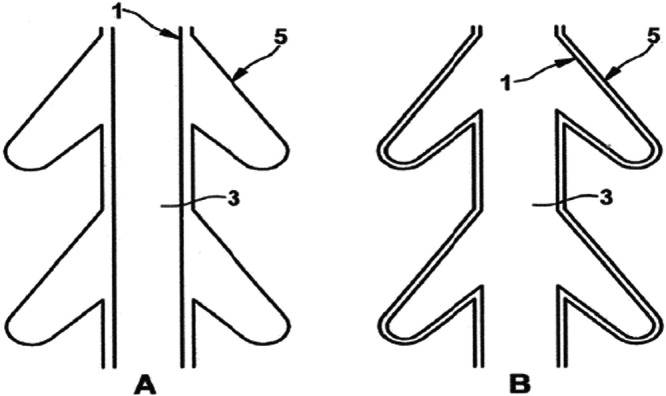
Cross‐section of the dental root canal model. (A) Endodontic point within the tooth model before ultrasonic welding (1) and polyisoprene to be liquefied (3). (B) Effective filling of polyisoprene after liquefaction to the limits of the simulated anatomy, with obturation completed after USW (5).

In addition to intracanal obturation, ex vivo evaluation of material behavior was performed using custom‐fabricated clear plastic obturator core models (types I and II), which mimicked lateral canal structures. Gutta‐percha prototypes (types I and II) were tested for flow dynamics using the same ultrasonic generator setup, and lateral flow distribution patterns were recorded (Figure 3).

### 2.7 Evaluation Methods

After obturation, the teeth were examined for structural changes. Micro‐CT scans were obtained before and after obturation to quantify the effectiveness of filling and sealing. Ex vivo evaluation of material behavior was performed using clear plastic obturator core models (types I and II), and lateral flow distribution patterns of gutta‐percha were recorded.

### 2.8 Micro‐Ct Evaluation and Metrics

Post‐obturation micro‐CT scans were reconstructed and analyzed using SkyScan NRecon and CTAn software (Bruker microCT). A 3D volumetric analysis was performed to quantify obturation quality. The total volume of the prepared root canal space was segmented. Within this volume, the filled material and unfilled voids were automatically differentiated using a global grayscale threshold. The primary metric was the void volume percentage, calculated as: (total void volume/total canal volume) × 100%. Secondary evaluation included a binary assessment of lateral canal penetration (present/absent) for all identifiable lateral canals in the pre‐obturation scan.

The primary metrics for obturation quality were the void volume percentage within the prepared canal and the lateral canal penetration rate. Apical extrusion was assessed as a distinct parameter. Extrusion was defined as the presence of obturation material extending > 0.5 mm beyond the apical foramen in the 3D reconstruction. Cases with extrusion were recorded but were not excluded from the primary sealing analysis. The volumetric void analysis was confined to the intracanal space.

### 2.9 Statistical Analysis

The data were analyzed using SPSS Version 26. Mean void reduction was calculated together with 95% confidence intervals (CI), and lateral canal penetration was computed as a percentage.

#### 2.9.1 Disclosure

The ultrasonic welding technique and customized gutta‐percha points described are covered by patents (US11,957,529B2, US10,898,295B1, SA14693) owned by the primary author.

### 2.10 Results

#### 2.10.1 Lateral Canal Penetration and Void Reduction

Compared with widely applied obturation methods such as warm vertical compaction and lateral condensation, the ultrasonic welding (USW) technique demonstrated greater control and enhanced thermoplastic flow. Lateral canal penetration was achieved in 87% of the samples (26/30 teeth; 95% CI: 79%–95%), indicating effective filling of accessory canals that are often missed with conventional techniques. The ultrasonic welding–assisted technique demonstrated a mean void reduction of 62% (95% CI: 58%–66%, *p* < 0.001). The results were consistent across both sample sets (*n* = 15 each), supporting the reproducibility and reliability of the technique. This reduction in voids suggests improved sealing quality, which may reduce the risk of bacterial leakage and enhance long‐term endodontic success.

#### 2.10.2 Temperature and Material Behavior

The maximum recorded temperature during ultrasonic activation was 44.2°C, remaining safely below the threshold for thermal damage to periapical tissues. The viscosity of the thermoplastic core decreased by 5%–25% during activation, facilitating better flow and adaptation within the canal system. Full resolidification occurred 3–6 s after deactivation, demonstrating rapid stabilization of the obturation material and efficient handling properties.

#### 2.10.3 Endo Ultrasonic Welding Obturation

**Figure 4:**
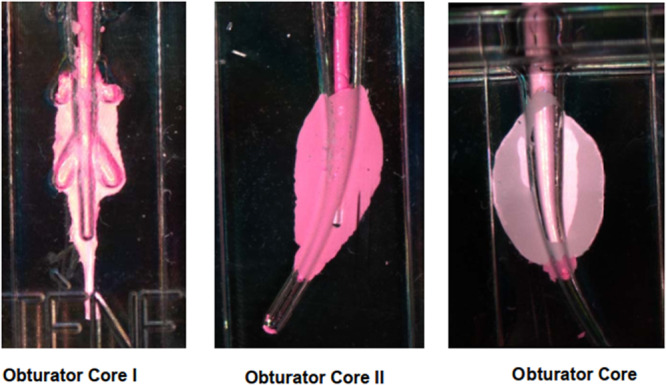
Obturator cores showing melted gutta‐percha with lateral flow resembling filling of lateral canals.

Results show melted gutta‐percha flowing sideways comparable to lateral canals (Figure 4).

**Figure 5:**
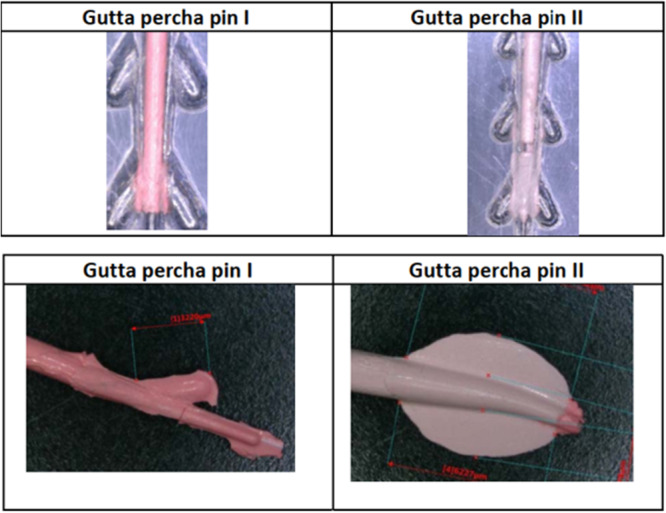
Melted gutta‐percha flowing sideways, comparable to lateral canals, after ultrasonic instrumentation.

The results of the tests with different types of ultrasonic instruments were positive, showing evidence of melted gutta‐percha flowing sideways, comparable to lateral canals. (Figure 5). Further explanation on the results of the experiment is available in the Supporting Information S1 and S2: Files 1 and 2.

**Figure 6:**
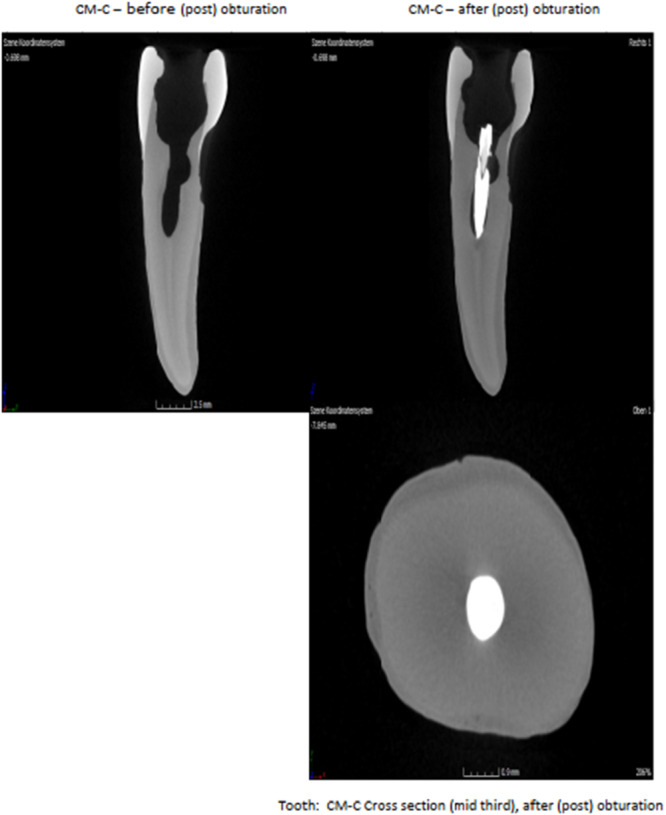
CT Images of teeth obturated.

**Figure 7:**
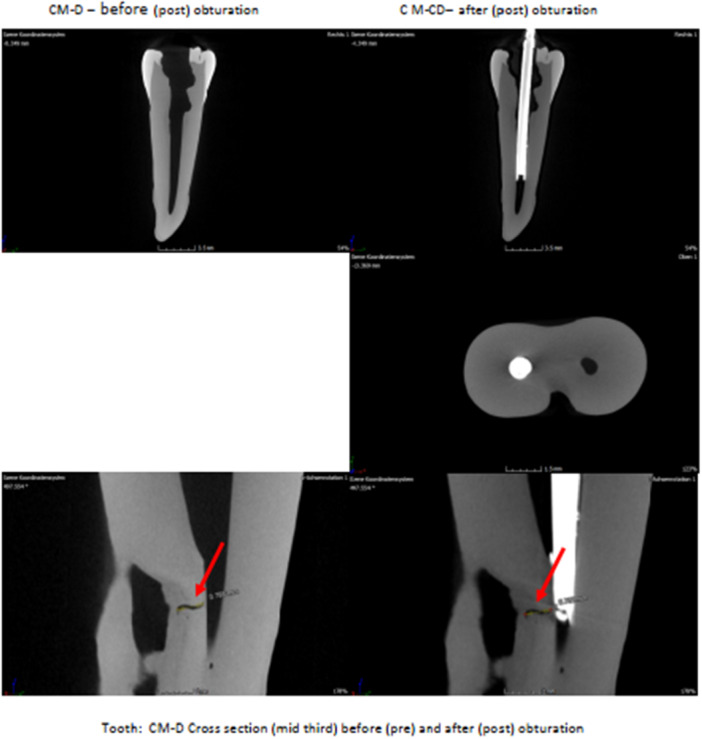
CT Images of teeth obturated. N.B.: Red arrows indicate tooth fractures before and after obturation. No microcrack propagation was observed.

Experiment to demonstrate that the persistent vibrations and/or mechanical energy have no fractural consequence on dentine and tooth structure (Figures 6 and 7).

### 2.11 Structural Integrity

Micro‐computed tomography (micro‐CT) imaging revealed no internal cracks or structural damage, confirming that the ultrasonic activation did not compromise tooth integrity. This finding supports the safety of the technique for ex vivo application and its potential for future clinical use.

### 2.12 Reproducibility

The second round of testing (*n* = 15; #16–30), performed by different researchers under identical experimental conditions, produced results consistent with those of the first sample set (*n* = 15). This demonstrates that the USW‐assisted obturation technique is reproducible across operators and sample sets, providing strong evidence of its reliability.

### 2.13 Quantitative Comparison With Conventional Methods

As summarized in Table 1, the USW technique outperformed the previous conventional obturation methods (Öztürk et al. 2024) in both void reduction and lateral canal penetration, highlighting its potential advantage in achieving a denser, more homogenous root canal filling (Table 1).

**Table 1:** Void reduction and lateral canal penetration level of ultrasonic welding‐assisted technique.

Group	Sample size (*n*)	Mean void reduction (%)	95% CI (void reduction)	Lateral canal penetration (%)
Ultrasonic welding	30	62	58–66	87 (26/30)

### 2.14 Demonstrations of Technique

To further illustrate the functionality and novelty of the ultrasonic welding‐assisted obturation technique, the patented design and mechanism are provided as Supporting Information S1.

## 3 Discussion

Gutta‐percha is a natural thermoplastic derived from the sap of a Southeast Asian tree, i.e., Palaquium gutta, that has been verified as biocompatible, thermoplastic, and radiopaque. It is considered the gold standard among endodontic filling materials (Dobrzańska et al. 2021). It is a polyturpene polymer of isoprene, which is a major component of natural rubber (Kim et al. 2021); however, unlike amorphous latex elastomers, gutta‐percha can crystallize to become a more rigid material. Gutta‐percha allows molding when heated, followed by hardening under cooling (Wang et al. 2025).

Our results conform with the hypothesis that ultrasonic energy can modulate the flow of gutta‐percha without compromising its structural integrity. The temperatures recorded are consistent with those obtained in prior studies, indicating that periodontal safety is ensured at temperatures below 45°C (9, 10). The USW technique promotes the use of gutta‐percha in root canal procedures since the superior liquidity achieved via this energy source enables the filling of even difficult spaces and addresses, therapeutically, any challenging canal anatomy.

The engineered behavior of the patented thermoplastic composition in the welding‐assisted obturation system allows transitions between phases in response to ultrasonic energy. This allows for controlled flow, enhanced adaptation, and three‐dimensional sealing, thereby surpassing the limitations of conventional heat‐based systems. Clinically, this technique meets the challenges of curved canals, irregular morphologies, or scenarios requiring minimally invasive approaches. The precise energy delivery and localized thermoplastic response reduce the risk of overfilling and enhance sealing in critical apical and lateral areas.

Recent studies in JOE have highlighted the limitations of traditional obturation techniques for complex anatomies: Only 60% lateral canal penetration with warm vertical compaction (Vazquez‐Alcaraz et al. 2024). Whereas our study revealed 87% penetration via USW‐assisted obturation (Kim et al. 2021), we noted that ultrasonic activation enhances single‐cone obturation, but our approach extends this benefit to thermoplastic flow, achieving a 50%–70% reduction in voids, thus offering a novel solution for three‐dimensional sealing (Yassen et al. 2011). Recent studies published in JOE (2021–2023) have also emphasized the need for enhanced obturation flow into the lateral anatomy, but no studies to date have reported a USW‐based mechanism. Thus, our work provides a novel progression in this area.

The fundamental basis of this study and comparative analysis is that compared with currently employed heat sources, USW offers the next‐level step in increasing the liquidity of gutta‐percha. When different ultrasonic instruments were applied to the HyFlex gutta‐percha pins, the results were consistent: Formation of lateral projections of gutta‐percha resembling anatomic lateral canal filling (i.e., migration of the fluid corresponding to the actual lateral canal anatomy). Enhanced adaptation to irregular canal walls. No evidence of structural damage to the dentinal tissues despite extended application periods (up to 6 s). Temperature readings within the biologically acceptable parameter range.

Notably, USW offers better fluidity for migration into voids while not otherwise fundamentally altering the steps of the procedure or introducing a substantial learning curve. The application of USW technology can reduce the clinical chair time by enabling faster and more precise obturation, improving patient comfort, and reducing the risk of procedural errors in challenging cases. Compared with warm vertical compaction, ultrasonic activation offers precise energy delivery, thereby avoiding the risks of overfilling or thermal necrosis. Additionally, the technique enables better penetration into accessory canals, which often remain unfilled via traditional methods (Liesegang et al. 2021). Our transparent obturator core models revealed practical results, namely, clear lateral extrusion of melted gutta‐percha during ultrasonic activation. Additionally, confirmation by subsequently implementing the same protocol in which the sample size was doubled (from *n* = 15 to *n* = 30) revealed consistent outcomes, i.e., reproducibility, which supports the reliability and validity of our results. These findings support the clinical potential of USW to enhance sealing in anatomically complex canals. The absence of fractures suggests safety, as continuous vibration does not exceed the critical thresholds shown in prior dental fracture models (Megha Patel et al. 2022).

### 3.1 Limitations of the Study

Limitations of this study include the use of bovine teeth (in vitro) instead of human teeth, which may limit the application of the results to clinical practice (please refer to Future Integration below in regard to human integration) However, increasing the sample size to 30 teeth (*n* = 30) reduced the variability in void reduction (from 40% to 75% to 50%–70%), enhancing the precision of the findings. Future studies should aim to assess long‐term effectiveness and patient comfort to address the traditional challenges of root canal procedures

This ex vivo model did not incorporate a simulated periodontal ligament for thermal dissipation, which may affect the extrapolation of temperature findings to a clinical scenario. Future studies will employ models with such simulations to obtain more conservative, clinically relevant thermal data. Additionally, while the technique is designed for efficiency, this study did not formally quantify the operative time compared to standard techniques like warm vertical compaction or single cone obturation. Furthermore, in clinical practice, the presence of significant canal curvature might reduce the efficiency of ultrasonic wave transmission along a metal plugger, potentially affecting the thermal profile and flow characteristics of the material in curved segments.

### 3.2 Future Integration of Patent Ultrasonic‐Activated Obturating Materials

This study is supported via a translational research pathway by three granted patents: (Patents 1) US11,957,529B2 (Alshehri 2024b), (Patents 2) US10,898,295B1 (Alshehri 2021); and (Patents 3) SA14693) (Alshehri 2024a). These patents describe endodontic points made of ultrasonic‐malleable thermoplastic materials. While enhanced canal adaptation and lateral penetration are followed by immediate resolidification, these patented technologies entail the use of thermoplastic or hybrid compositions, e.g., gutta‐percha derivatives, bioactive silicone polymers, and reversible phase‐change carriers, of which the viscosity can be temporarily reduced via ultrasonic activation. Future phases will focus on material miniaturization, delivery optimization, and validation under clinically simulated human conditions.

## 4 Conclusion

Effective filling and sealing are the essential conditions of the root canal procedure, so any improvement, either theoretical or actual, should be pursued in advancing science. USW‐assisted obturation introduces a transformative approach that merges mechanical energy with advanced biomaterials, potentially increasing endodontic outcomes by enhancing interface adaptation and minimizing procedural errors.

This proof‐of‐concept study demonstrated that USW‐assisted obturation significantly increased material flow, canal adaptation, and sealing efficiency without inducing harmful thermal, biological, or mechanical effects. Despite the lack of data that would allow projections of its long‐term effectiveness, the USW technique exhibits high potential for integration into clinical endodontic practice, justifying further study. The combination of USW technology with the conventional use of gutta‐percha, which increases the effectiveness of this procedure, is logical, given our results. This technique, if validated clinically, could shift the paradigm in modern endodontic obturation via the integration of precision energy control with material responsiveness. Future work should incorporate tests (e.g., thermal cycling, pH cycling) to simulate oral environmental challenges and assess sealing durability. Future research should also include a controlled clinical trial to objectively measure the comparative operative time and evaluate the technique's efficiency in a clinical setting.

## Author Contributions

**Mohammed Alshehri** conceived the study, developed the ultrasonic welding–assisted obturation concept and patented materials, designed and supervised all experimental phases, analyzed and interpreted the data, and drafted the original manuscript. **Chaniotis Antonis** contributed to the experimental design, provided endodontic expertise, assisted with data interpretation, and critically revised the manuscript. **Hadi Alamri** participated in ex vivo experimentation, data acquisition, and micro‐CT analysis and contributed to manuscript revision. **Ahmad AlQahtani** assisted with sample preparation, experimental procedures, and data validation, and reviewed the manuscript. **Mohammed Altamimi** contributed to methodological optimization, interpretation of findings, and critical revision of the manuscript. All authors reviewed and approved the final version of the manuscript and agree to be accountable for all aspects of the work.

## Conflicts of Interest

The primary author is the inventor and owner of the patents related to the ultrasonic welding technique and obturation materials used in this study (US11,957,529B2, US10,898,295B1, SA14693). All authors declare no competing interests.

## Patent

The materials and applicator design used in this study are protected under a granted US patent (US11,957,529 B2), reinforcing the originality and translational readiness of this technique for future clinical application.

## Supporting information

Supporting File 1

Supporting File 2

## Data Availability

The data supporting the findings of this study are available from the corresponding author upon reasonable request.
