# Supplementary material for: A Novel Ultrasonic Welding‐Assisted Thermoplastic Root Canal Obturation Technique: An Ex Vivo Proof‐of‐Concept Study
Source: Clin Exp Dent Res. 2026 Apr 17;12(2):e70356. doi: 10.1002/cre2.70356 (PMC13088893; doi:10.1002/cre2.70356)
Supplement: Supplementary file 1 — Supporting File 1 [file CRE2-12-e70356-s001.pdf]

Exploratory Study

Endo Ultrasonic Welding Obturation

Part II

Copy to:

MS

Table of contents

1. Introduction 3
2. Objectives 3
3. Indication 3
4. Principles of Ultrasonic Welding (USW) 3
4.1. Definition of USW 3
4.2. Ultrasonic Welding Equipment 4
4.3. Components of USW 4
4.4. Description of USW 5
5. Motivation & Hypothesis 5
6. Methodology 5
7. Results 6
7.1. Theoretical results 6
7.2. Practical results 6
8. Conclusion 7
9. Further steps 7
10. Literature 7

Endo Ultrasonic Welding Obturation II

1. Introduction

This current report presupposes the contents of the report of Endo Sonic Welding_Summary/Part I. And it summarises the status of the exploratory project, describing the activities carried out so far and the corresponding results.

2. Objectives

The aim of the project is to explore the possibility of using ultrasonic energy in melting gutta percha and to use the technology in the obturation of root canal.

3. Indication

Fast and efficient permanent root canal obturation

4. Principles of Ultrasonic Welding (USW)

4.1. Definition of USW

Ultrasonic Welding (USW) is a welding technique that uses high frequency ultrasonic vibration energy to weld two pieces of materials together

4.2. Ultrasonic Welding Equipment

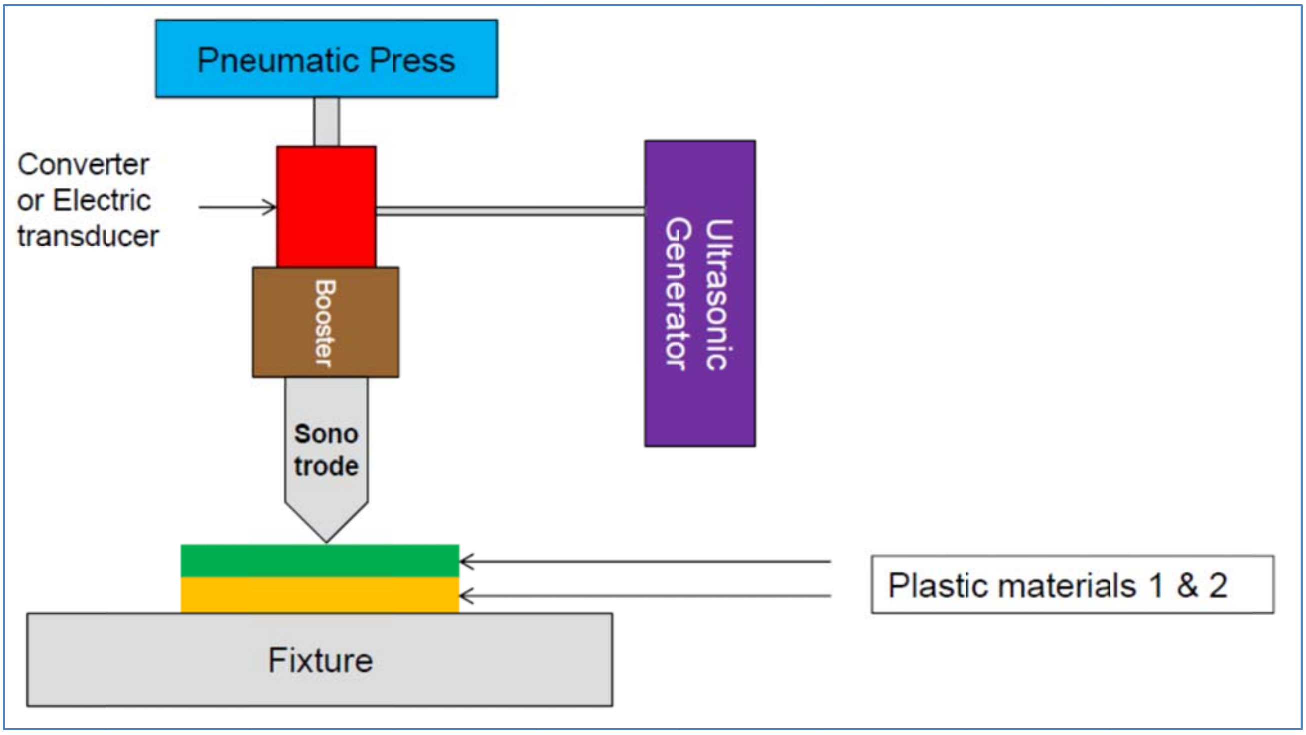

4.3. Components of USW

- An Ultrasonic Generator
- An Ultrasonic Stack System
  - converter or piezoelectric transducer
  - a booster
  - and a sonotrode or horn
- A press
- Fixture: Clamping device

4.4. Description of USW

- **Ultrasonic Generator:** generates and delivers high frequency electrical signal the stack system
- **Ultrasonic Stack System**
  - Converter: converts the electrical signals into high frequency mechanical vibration through Piezoelectric effect
  - Booster: modifies the amplitude of vibration mechanically
  - Sonotrode or Horn: vibrates at high frequency and transmits the mechanical vibration to the two pieces to be welded
- **Press:** Applies pressure on the two plastic pieces to be joined
- **Fixture:** clamping device which is used to hold and clamp the two plastic pieces together

5. Motivation & Hypothesis

The underlying hypothesis is to utilise and apply high frequency vibrating ultrasonic energy onto a thermoplastic material for example gutta percha, under pressure to generate frictional heat at the thermoplastic (gutta percha) joint surface.

All things being equal, the gutta percha would melt, flows into the accessory canals and bond to the dentinal surface, when the vibration energy is removed.

6. Methodology

Determination of the following parameter:

- Core material and coating material
- Ultrasonic frequency
- Power
- Amplitude
- Cooperation partner

7. Results

7.1. Theoretical results

- Polymer core material and coating gutta percha material were defined
- Working frequency and some other parameters were defined
- Values of Power and Amplitude are estimated
- NDA was signed with a likely cooperation partner

7.2. Practical results

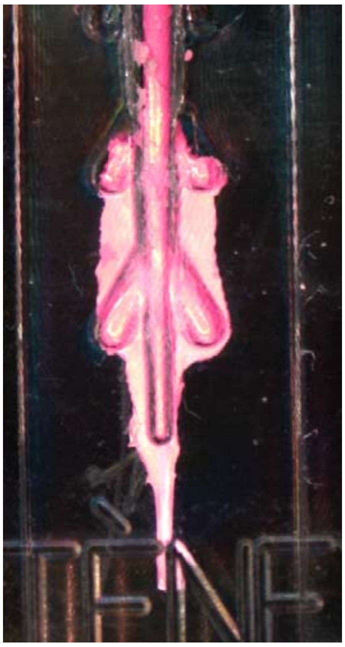

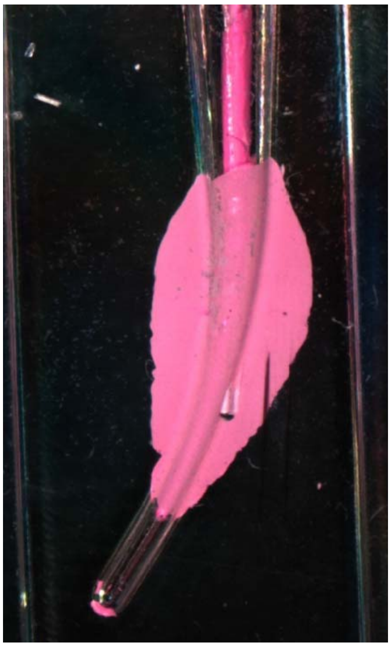

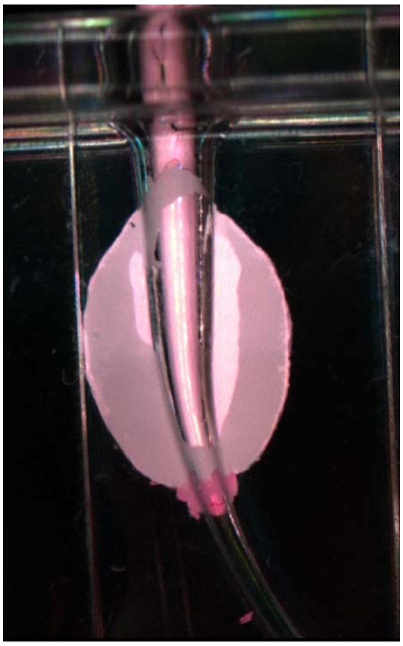

Obturator Core I **Obturator Core II** **Obturator Core**

Results show melted gutta percha flowing side ways comparable to lateral canals

8. Conclusion

The technology exists already in different fields, e.g. Wood welding, Medical, Automotive, Electronics, Consumer products, Toys, Packaging and Appliance. It is new in dentistry.

Results of the investigation during the exploratory stage are good and promising. Therefore, based on the investigation so far, we think that the project could be realised, unless certain circumstances beyond our control is encountered during the development stage, since we are working in a micro level and lots of challenges should be anticipated.

However, whether there is any risk in dental application or not is unknown and not explored, especially in endodontics. This will have to be checked, before the exploratory project is moved into the feasibility stage.

9. Further steps

Determine and explore all likely risks that may arise in conjunction with the technology in endodontic application, for instance, temperature increase in the tooth, fracture of tooth due to vibrational force, patients' perception of the frequency during treatment etc. In addition, the cost of device and overall project cost would have to be estimated.

10. Literature

1. https://science.howstuffworks.com/ultrasonic-welding1.htm
2. Kaul S, Design and Analysis of Sonotrode for Ultrasonic Eye-Punching Process
3. Alvin Goodman, Thermoproperties of GP
4. Long Yu et al, Polymer blend and composites from renewable resources
5. XU C, Physical blend of PLA_NR
6. Syed Farhan razan, Ultrasonic Welding of Thermoplast_Thesis
