# Supplementary material for: A Novel Ultrasonic Welding‐Assisted Thermoplastic Root Canal Obturation Technique: An Ex Vivo Proof‐of‐Concept Study
Source: Clin Exp Dent Res. 2026 Apr 17;12(2):e70356. doi: 10.1002/cre2.70356 (PMC13088893; doi:10.1002/cre2.70356)
Supplement: Supplementary file 2 — Supporting File 2 [file CRE2-12-e70356-s002.pdf]

Exploratory Study

Endo Ultrasonic Welding Obturation

Part III

Endo Ultrasonic Welding Obturation III

Table of contents

1. Introduction 3

2. Objectives and Indication 3

3. Methodological approach 3

3.1. Address the following subject matters: 3

4. Achievements and Experiments 4

4.1. Streamline the specification 4

4.2. Further tests carried out to support primary results obtained previously 4

4.3. Risks that could arise as a result of the use of ultrasonic technology in endodontics 4

4.4. Experiment to demonstrate that the persistent vibrations and/or mechanical energy of the ultrasonic instrument have no fractural consequence on dentine and tooth structure 4

4.5. Technical evaluation and search for a cooperation partner for the production at a maximum COG of 600 € 5

4.6. Re-evaluate the patent issue 5

5. Results 5

5.1. Fig 1. Results of the tested ultrasonic instruments and corresponding Gutta percha pin I. 5

5.2. Risk analysis that could arise as a result of the use of ultrasonic technology in endodontics 6

5.2.1 Table 1 Probable risks defined as a result of the application of USW in endodontics 6

5.3. Experiment to demonstrate that the persistent vibrations and/or mechanical energy have no fractural consequence on dentine and tooth structure 7

5.3.1 Fig 2. CT Images of teeth obturated 7

.... 11

5.4. Technical evaluation and search for a cooperation partner for the production at a maximum COG of 600 €. 13

6. Conclusion 13

7. Further steps and recommendations 13

8. Literature 13

Endo Ultrasonic Welding Obturation III

1. Introduction

The current like all the previous reports, presupposes the contents of the report of Endo Ultrasonic Welding Summary/Part II. And it summarises the status of the exploratory project, describing the activities carried out so far and the corresponding results.

Based on the last report and in accordance with the demands of the ideation team review meeting, to "prepare a list with open topics (temperature increase, cost of device…), which have to be considered during development", we determined and explored all likely risks that might arise in conjunction with the technology during endodontic application. Some of these risks for instance are temperature increase in the tooth, fracture of tooth due to vibrational force, patients' perception of the high frequency during treatments etc. In addition, the cost of device and overall project cost was estimated.

2. Objectives and Indication

The aim of the project is to explore the possibility of using ultrasonic energy in melting gutta percha and to use the technology in the obturation of root canal.

3. Methodological approach

3.1. Address the following subject matters:

- Streamline the specification
- Further experiments as proof of concept
- Define risks that could arise as result of the use of ultrasonic technology in endodontics
- Prove and demonstrate that the persistent vibrations and/or mechanical energy of the ultrasonic instrument have no fractural consequence on dentine and tooth structure
- Technical evaluation and search for a cooperation partner for the production at a maximum COG of 600 €
- Re-evaluate the patent issue

4. Achievements and Experiments

4.1. Streamline the specification

Approximation of the specification was streamlined

4.2. Further tests carried out to support primary results obtained previously

More experiments were carried out to demonstrate in a rudimentary sense the proof of concept and support the previous results obtained. Refer in addition to the report of Endo Ultrasonic Welding Summary/Part II.

In a brief description, the “Gutta Percha Pins” (HyFlex Gutta Percha Pins) were inserted into plastic root canals. After which the tips of the different sonotrodes were placed onto the head of the pins. And they were obturated with the ultrasound device (different ultrasonic instruments), activated for approximately 2 - 8s depending on the type of ultrasonic device employed. The devices differed in their mode of actions and power. Therefore, there were differences in the melting capability of the gutta percha.

4.3. Risks that could arise as a result of the use of ultrasonic technology in endodontics

Probable risks that could arise as a result of the use of ultrasonic technology in endodontics and the consequent hazards were defined. The risks are listed in section of 5.2.)

4.4. Experiment to demonstrate that the persistent vibrations and/or mechanical energy of the ultrasonic instrument have no fractural consequence on dentine and tooth structure

In order to proof that the persistent vibrations and/or mechanical energy of the instrument have no fractural consequence on dentine and tooth structure, the following in vitro test was conducted.

Selected bovine teeth were decoronated and the roots were prepared with HyFlex CM and HyFlex EDM respectively. After irrigation and drying, the root canals were obturated with the ultrasonic device. CT images of the teeth were taken before and after the preparation and final obturation.

During the obturation, the ultrasonic vibration was intentionally applied for a longer time (6s) than necessary for the obturation in order to check whether the vibrations/mechanical hammering caused damage to the tooth structure and whether the gutta percha pin melted due to the heat development. And at the same time the temperature on the surface of the teeth was determined.

The temperature on the surface of the teeth was measured with Infra red thermometer, while melting of gutta percha and crack development or propagation was detected wit computer tomography.

4.5. Technical evaluation and search for a cooperation partner for the production at a maximum COG of 600 €

In order to fulfil the demands of the ideation committee, that the COG (Cost of good) product should not exceed EUR 600.- and check whether it is technically feasible, we searched for corresponding suppliers.

4.6. Re-evaluate the patent issue

Patent issue was also re-addressed, and patent search was expanded.

The patent of Dr. Mohammed Alshehri could not be retrieved on line. However, a draft was received from him. But this could not help further.

The complete information regarding the registration and filing is necessary to guarantee the securing of the priority right. Otherwise, it will hinder contacting of third party contractors, especially in China.

5. Results

5.1. Fig 1. Results of the tested ultrasonic instruments and corresponding Gutta percha pin I

Gutta percha pin I	Gutta percha pin II
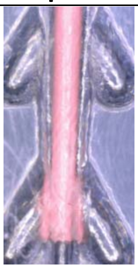	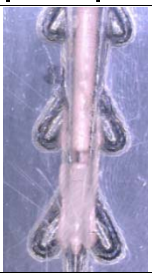

Gutta percha pin I	Gutta percha pin II
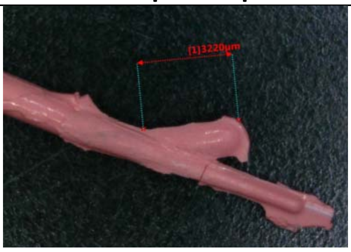	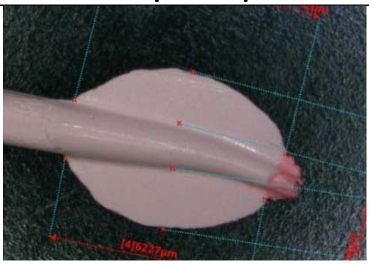

Results of the tests with different types of ultrasonic instruments were positive. There was evidence of melted gutta percha flowing side ways comparable to lateral canals.

5.2. Risk analysis that could arise as a result of the use of ultrasonic technology in endodontics

In the following table, we summarise the probable risks that could arise as a result of the use of ultrasonic technology in endodontics and the consequential hazards. An evaluation under the column designated with “comment" was made, to support or not the probability of the occurrence of such risks.

5.2.1 Table 1 Probable risks defined as a result of the application of USW in endodontics

Probable Risks Defined as a Result of the Application of USW in Endodontics

Risk Description	Hazard	Comment	Literature
High Temperature: Increase in temperature due to the conversion of mechanical energy into heat energy at the dentine/gutta percha interface	Damage on dentine and surounding tissues (i.e., the periodontal ligament)	According to literature temperature within Canal in warm vertical compaction is less than 45°C (< 45°C), in endodontically treated teeth with two heating sources: System B Obtura (Analytic Technology, Redmond, WA, USA) and System MB (Shibrun Medical s.r.l, FE, Coronella, Italy)	Simeone M et al,Temperature Profiles Along the Root with Gutta-percha<br>* Further Literatures are: 2, 3, 4, 5
Tooth fracture due to continuous hammering of vibrational/mechanical energy on the surface of the GP material and indirectly on the tooth	Tooth fracture,	*According to the experiment conducted in our laboratory, no cracks were obtained<br>**And also in Maxillary surgery no facial fractures were reported	*See the results in the laboratory directory<br>**6. 90-421-02-07_06_15_SonicWeld_Basics<br>7. Aldana, P. R., et al.,<br>Ultrasound-aided fixation of a biodegradable cranial fixation system: uses in pediatric neurosurgery. J Neurosurg Pediatr, 3(5), 2009, S. 420-424
Accoustic Hearing Sound level/ intensity	Hearing loss	Danger only possible till > 20KHz (scientifically proven).<br>Or at a sound level and intensity over 100 dB.<br>Our working frequency would be are at a minium of 28 KHz<br>**High frequeny sounds >80dB (threshhold is headache, tinnitus, fatigue, dizziness and nausea, and dangerous for > 120 dB	8. Ultrasound and high frequency sound: www.belgium.be<br>9. Assistant Prof. Matthias Möbius, Hearing, Sound Intensity and Sound Level
Exposure to radiation	Non-Invasive Treatment	Non	

5.3. Experiment to demonstrate that the persistent vibrations and/or mechanical energy have no fractural consequence on dentine and tooth structure

It should be noted that the preparation of the teeth with HyFlex EDM and HyFlex CM respectively, was carried out by a dental technician who had no experience with teeth and root canal preparation. In fact, that was his first root canal preparation ever. Therefore, the canals were not uniformly prepared and could explain why there were discrepancies in the canal areas obturated, as the CT images show below.

CM, EDM refer to the file used in the preparation and B,C,D,G,H and I refer to tooth identification letter.

5.3.1 Fig 2. CT Images of teeth obturated

CM-B – before (pre) obturation CM-B – after (post) obturation

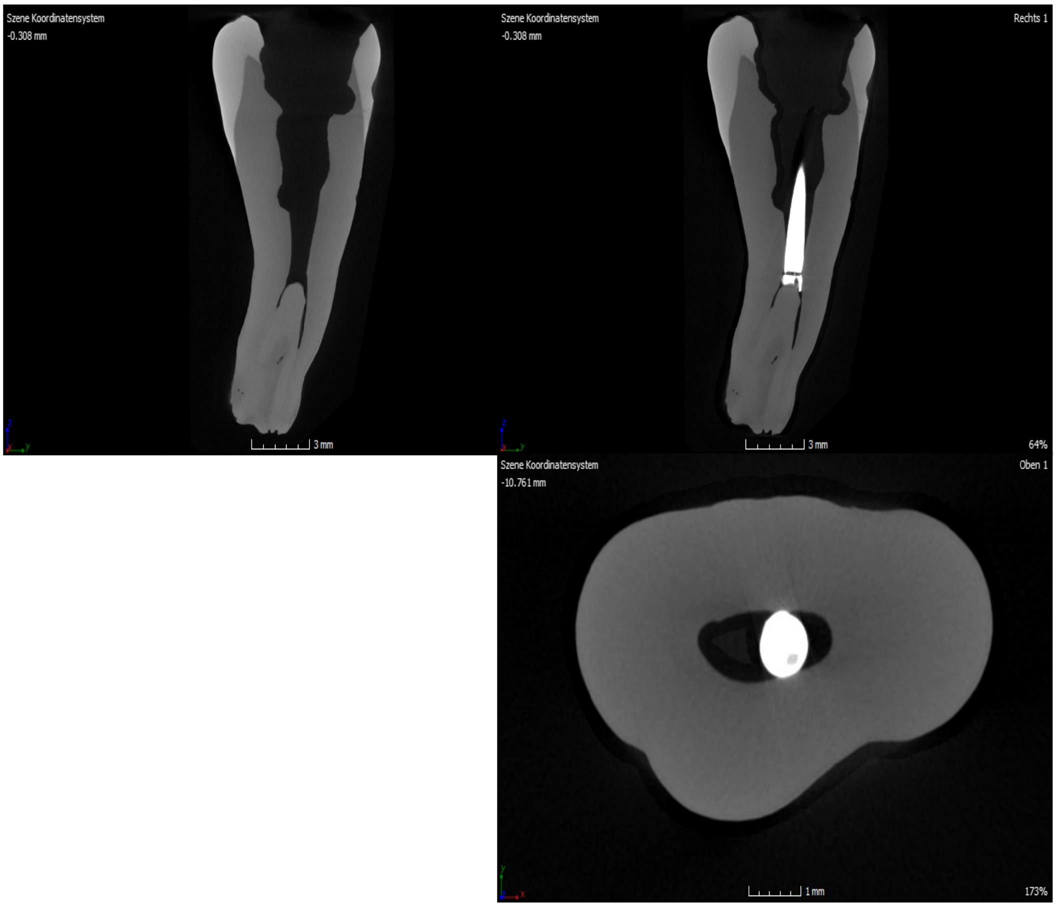

Tooth: CM-B Cross section (mid third), after (post) obturation

CM-C – before (post) obturation

CM-C – after (post) obturation

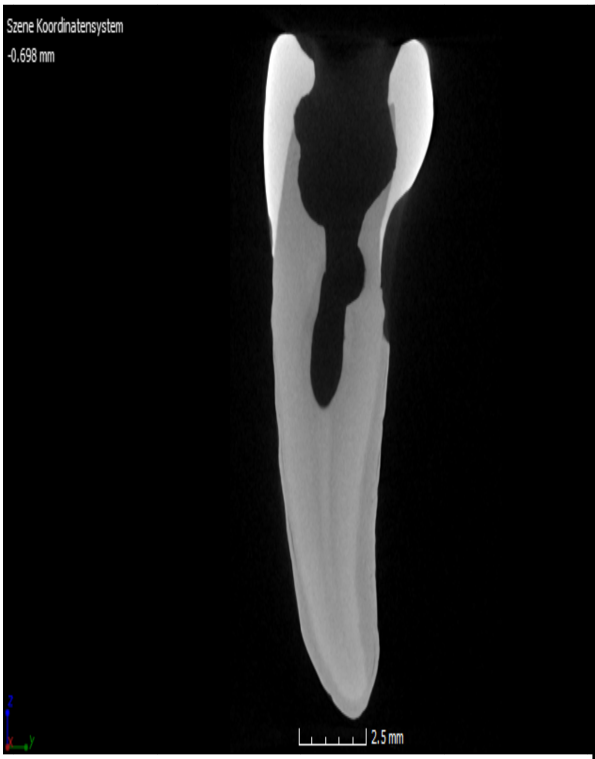

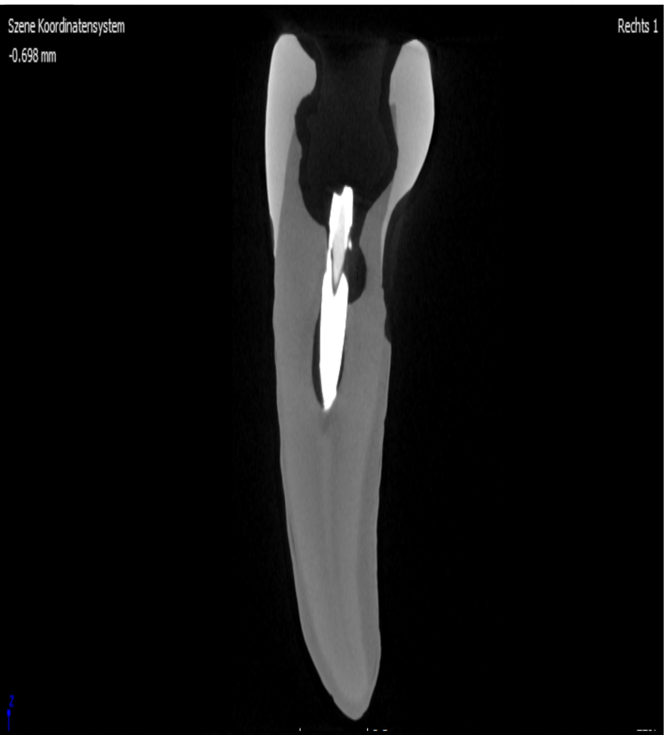

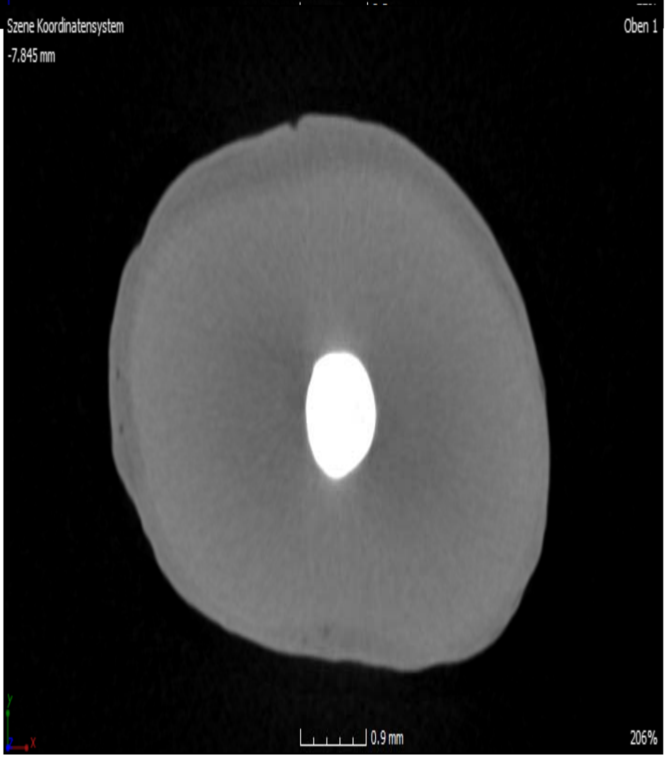

Tooth: CM-C Cross section (mid third), after (post) obturation

CM-D – before (post) obturation

C M-CD– after (post) obturation

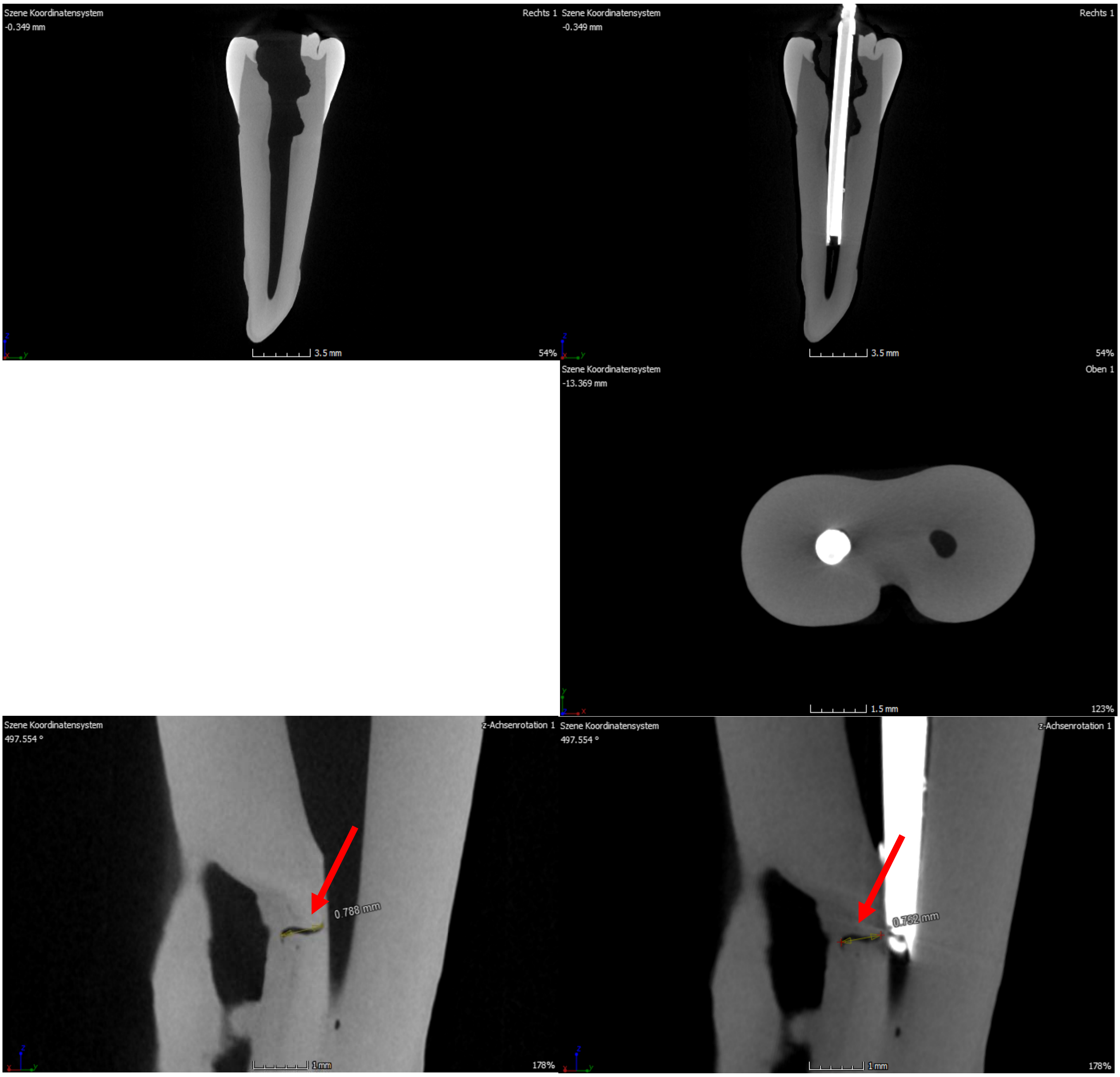

Tooth: CM-D Cross section (mid third) before (pre) and after (post) obturation

N.B. Red arrows depict tooth fracture before and after obturation. The was no micro crack propagation

EDM-G – before (post) obturation

EDM-G – after (post) obturation

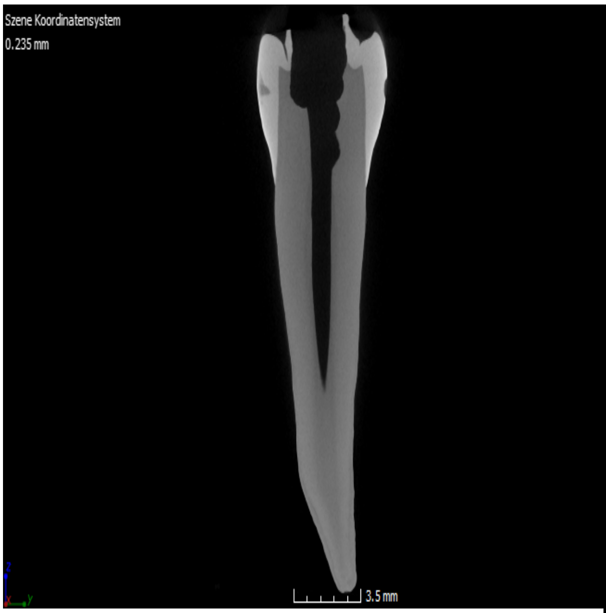

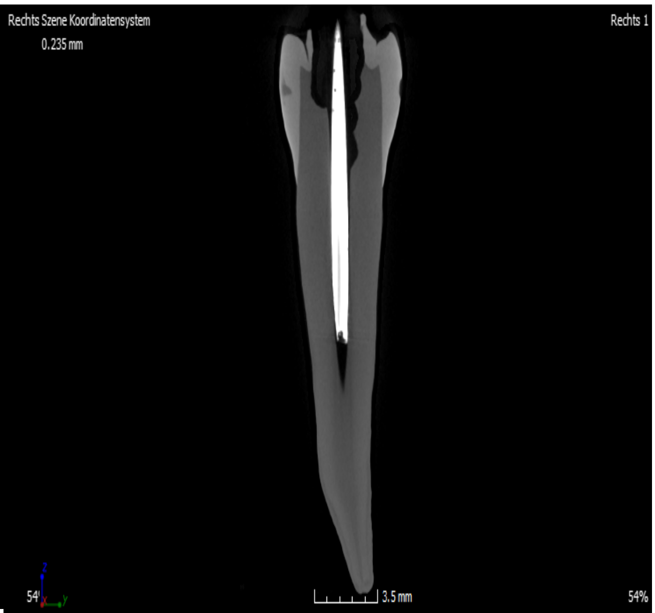

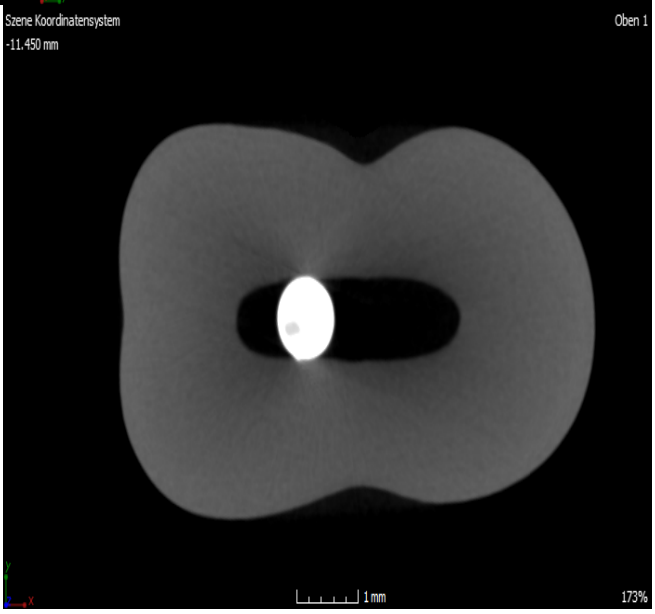

Tooth: EDM-G Cross section (mid third) after (post) obturation

EDM-H before (post) obturation

EDM-H after (post) obturation

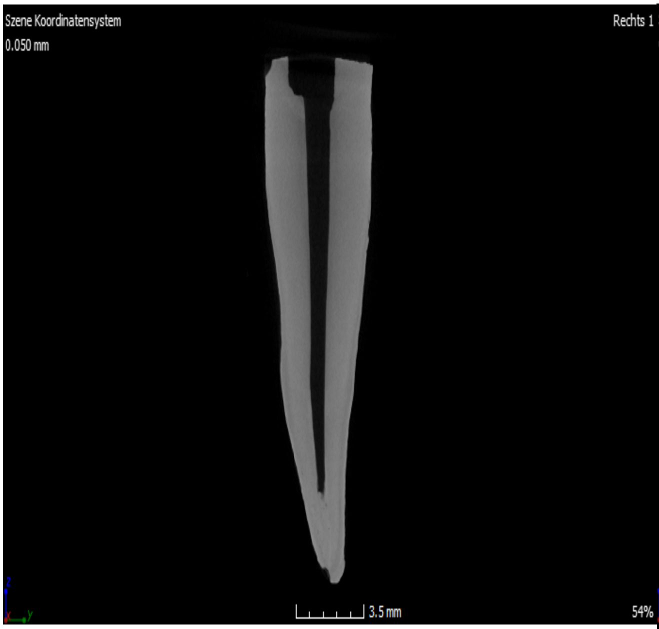

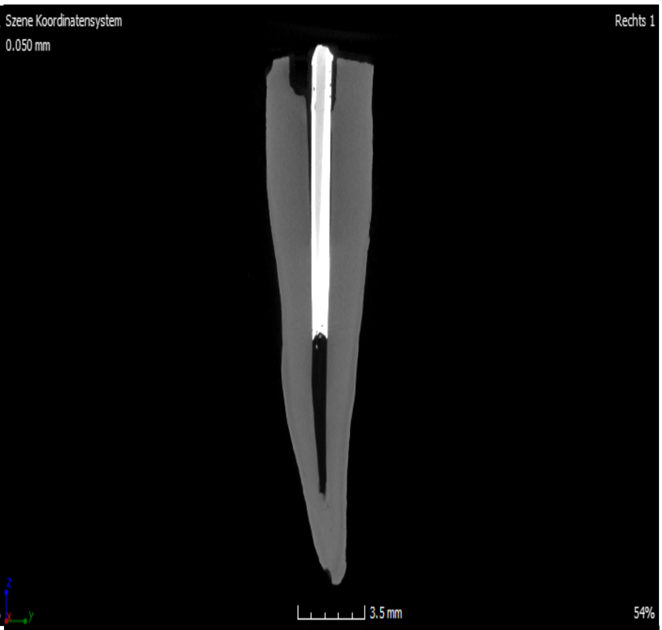

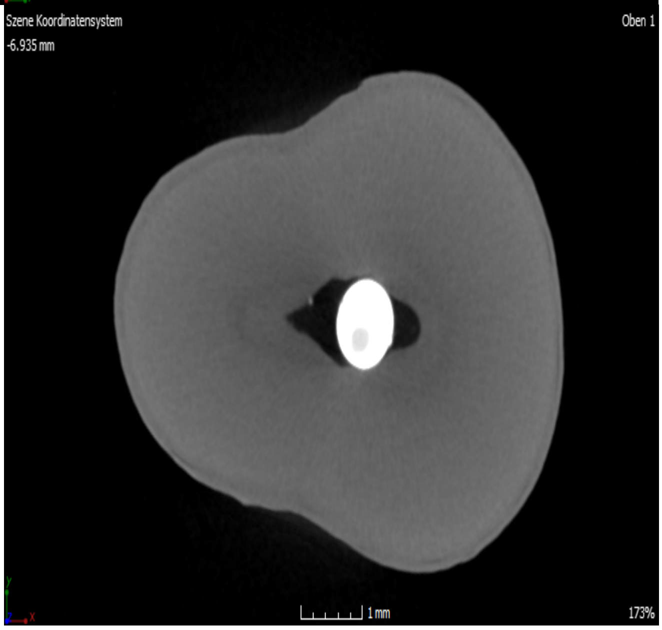

Tooth: EDM-H Cross section (mid third), after (post) obturation

EDM-I before (post) obturation

EDM-I after (post) obturation

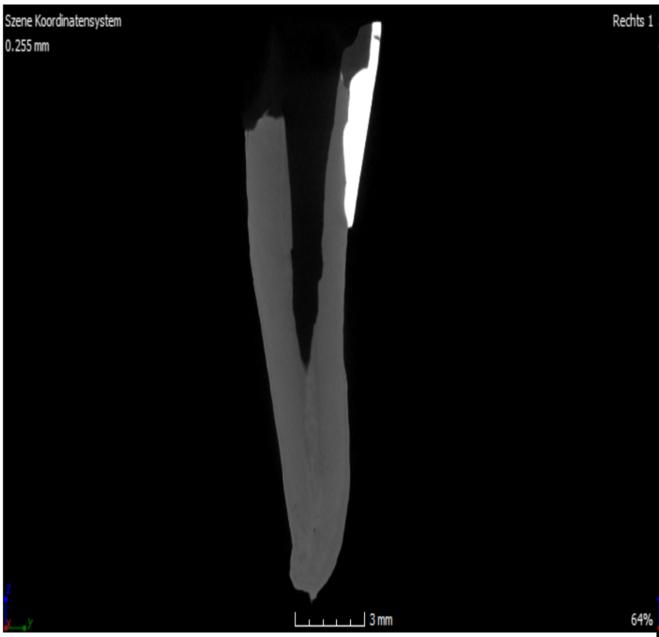

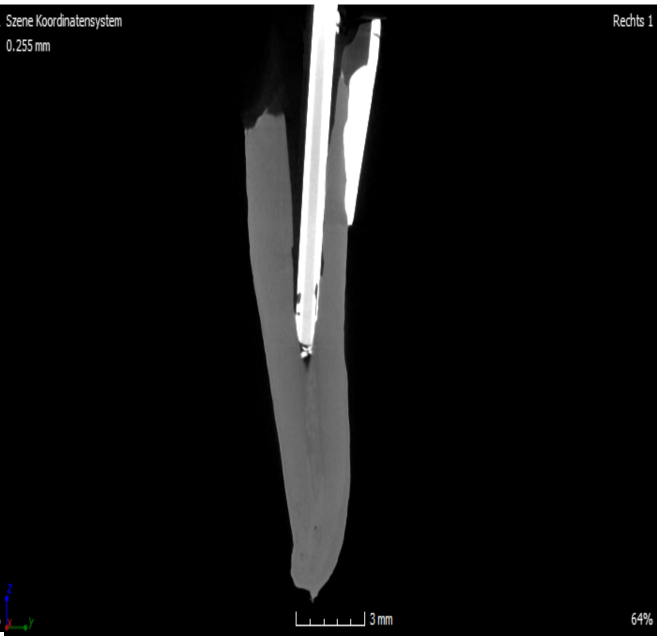

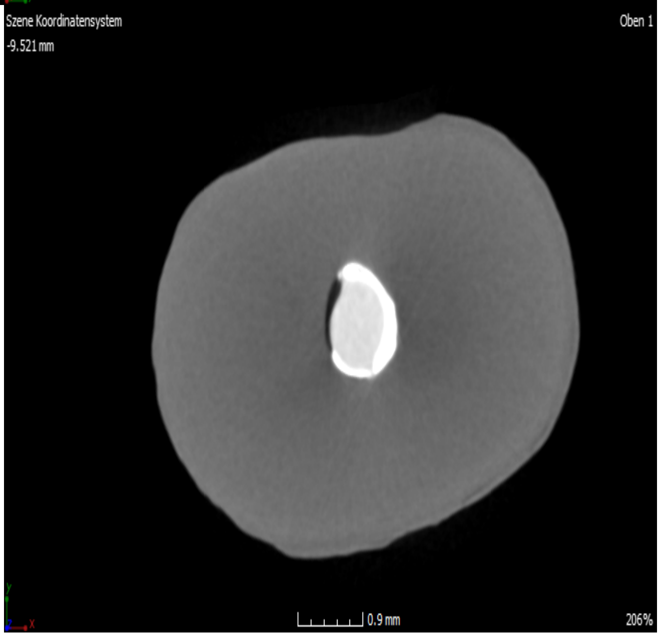

Tooth: EDM-I Cross section (mid third), after (post) obturation

With reference to the tooth CM-D, the length of the fracture/crack found on the tooth was measured with CT before and after the preparation and obturation respectively. As can be seen from the images, there was no evidence of further fracture or micro-crack propagation.

5.4. Technical evaluation and search for a cooperation partner for the production at a maximum COG of 600 €.

Tests performed sofar demontsrated that the technical realisation is very feasible. And a maximum COG of € 500 – 600.- could be achieved.

6. Conclusion

The results obtained from the investigation during the exploratory stage so far were positive and promising. Based on these investigations, we think that the project could be realised, unless certain circumstances beyond our control are encountered during the development stages, since we are working on a micro level and lots of challenges should be anticipated. These risks however are minimal, because as can be seen from the above results, experiments were carried out already in vitro on a micro level.

Clarification of the patent issue in Saudi Arabia would be very necessary before we can contact companies in China for the production of the device for a maximum COG of € 500 - 600.

7. Further steps and recommendations

- Patent issues should be looked into again, since the cat would be out of the bag soon. Otherwise, the right to the idea could be lost or stolen
- Cross-check and work with patent submitted in Saudi Arabia and think of PCT submission
- Move project officially to the next stage
- Define registration strategy, eventually with potential supplier
- Contact universities and centers that could perform a pre-clinical testing (Animal study) before clinical studies could be performed

8. Literature

1. See directory
